# CXCL10-Mediates Macrophage, but not Other Innate Immune Cells-Associated Inflammation in Murine Nonalcoholic Steatohepatitis

**DOI:** 10.1038/srep28786

**Published:** 2016-06-28

**Authors:** Kyoko Tomita, Brittany L. Freeman, Steven F. Bronk, Nathan K. LeBrasseur, Thomas A. White, Petra Hirsova, Samar H. Ibrahim

**Affiliations:** 1 Division of Gastroenterology & Hepatology, Mayo Clinic, Rochester, Minnesota 55905, USA; 2 Department of Physical Medicine & Rehabilitation Mayo Clinic, Rochester, Minnesota 55905, USA; 3 Division of Pediatric Gastroenterology and Hepatology, Mayo Clinic, Rochester, Minnesota 55905, USA.

## Abstract

Nonalcoholic steatohepatitis (NASH) is an inflammatory lipotoxic disorder, but how inflammatory cells are recruited and activated within the liver is still unclear. We previously reported that lipotoxic hepatocytes release CXCL10-enriched extracellular vesicles, which are potently chemotactic for cells of the innate immune system. In the present study, we sought to determine the innate immune cell involved in the inflammatory response in murine NASH and the extent to which inhibition of the chemotactic ligand CXCL10 and its cognate receptor CXCR3 could attenuate liver inflammation, injury and fibrosis. C57BL/6J *CXCL10*^−/−^*, CXCR3*^−/−^ and wild type (WT) mice were fed chow or high saturated fat, fructose, and cholesterol (FFC) diet. FFC-fed *CXCL10*^−/−^ and WT mice displayed similar weight gain, metabolic profile, insulin resistance, and hepatic steatosis. In contrast, compared to the WT mice, FFC-fed *CXCL10*^−/−^ mice had significantly attenuated liver inflammation, injury and fibrosis. Genetic deletion of CXCL10 reduced FFC-induced proinflammatory hepatic macrophage infiltration, while natural killer cells, natural killer T cells, neutrophils and dendritic cells hepatic infiltration were not significantly affected. Our results suggest that *CXCL10*^−/−^ mice are protected against diet-induced NASH, in an obesity-independent manner. Macrophage-associated inflammation appears to be the key player in the CXCL10-mediated sterile inflammatory response in murine NASH.

Nonalcoholic fatty liver disease (NAFLD) is an emerging public health problem linked to the alarming growth in the prevalence of obesity worldwide^1^. NAFLD is the most common liver disease; up to 46% of the U.S. population is affected, and up to 12% have the more inflammatory and progressive form of the disease termed nonalcoholic steatohepatitis (NASH)^2^,^3^. In addition to steatosis, NASH is characterized histologically by hepatocyte apoptosis^4^, hepatic infiltration by inflammatory cells and fibrosis^5^. Patients with NASH may progress to cirrhosis and its sequelae of end stage liver disease and hepatocellular carcinoma^6^,^7^. Indeed NASH is predicted to be the leading cause of liver transplantation in adults by 2030^8^. Aside from life style modification, NASH is still lacking effective regulatory agency approved pharmacotherapy. Hence there is a critical need to better define the cellular and molecular mechanisms that promote liver injury and inflammation in NASH, in order to identify specific therapeutic targets.

NASH is a lipotoxic disorder where high serum levels of saturated fatty acid (SFA)s induce liver injury^9^. In addition to liver cell injury, hepatic lipotoxicity is associated with inflammatory infiltrates which further promote liver injury and fibrogenesis^10^,^11^. We previously reported, largely by using *in vitro* studies, that the activated stress kinase mixed lineage kinase 3 (MLK3) mediates the release of C-X-C motif chemokine 10 (CXCL10)-enriched extracellular vesicles (EV)s from hepatocytes under lipotoxic conditions. These CXCL10-loaded EVs are potently chemotactic for cells of the innate immune system^12^. Therefore, CXCL10 on the EVs appears to play a pathogenic role in NASH.

CXCL10 is well known in hepatitis C as a hepatocyte-derived chemotactic ligand and initiator of inflammatory cascades via its cognate receptor C-X-C motif receptor 3 (CXCR3), which is widely expressed on multiple cells of the innate immune system, including Kupffer cells (the resident hepatic macrophages)^13^, dendritic cells^14^, natural killer (NK) cell, NKT cells^15^ and neutrophils^16^. Hence, these entire different innate immune cells are potential targets for CXCL10-mediated chemotaxis.

Many of these innate immune cells are implicated in NASH pathogenesis. For instance, intrahepatic dendritic cells (DC)s expand in NASH liver and assume an activated immune phenotype. However, DCs may also play a salutary role in NASH by limiting the sterile inflammation through clearing apoptotic cells and necrotic debris^17^. In addition, neutrophil accumulation is also a prominent feature of the inflammation observed in NASH, which induces tissue damage through generation of oxidants, mediated, in part, by their myeloperoxidase (MPO)^18^. Furthermore, mice fed an obesity-inducing diet display natural killer (NK) T cell apoptosis, reduced liver NKT cell populations, and excessive hepatic production of TNFα and interferon gamma promoting hepatic inflammation^19^. Macrophages have, also been implicated in NASH pathogenesis where an influx of circulating monocytes into the liver and their differentiation and polarization into proinflammatory macrophages has been documented^20^. Hence multiple cell types of the innate immune cells may participate in the hepatic tissue inflammation occurring in NASH. The strength of their contribution and the hierarchal relationships between them remains unclear.

Herein, we report a protective effect of CXCL10 genetic deletion in a murine model of diet-induced NASH. Specifically, we demonstrate reduction of liver inflammation and subsequent liver injury and fibrosis in CXCL10-null mice, independent of effects on weight gain and parameters of the metabolic syndrome. We also profiled innate immune cell populations involved in the sterile inflammatory response in our NASH mouse model, and observed only significant CXCL10-dependent reduction of hepatic proinflammatory (M1 polarized) macrophages accumulation and activation, when compared to other innate immune cells involved in the hepatic inflammation in NASH. Finally, we validate the important role of CXCL10 by studying mice deficient for its cognate receptor CXCR3 and demonstrate that these mice are also protected against diet-induced NASH.

## Results

### The metabolic phenotype of CXCL10^−/−^ mice

**Figure 1:**
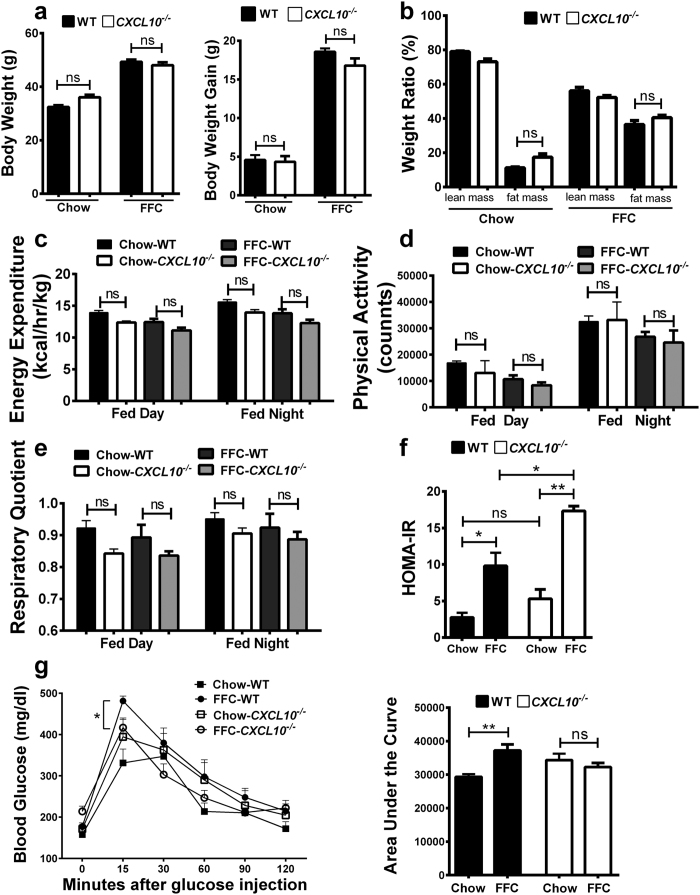
Metabolic phenotype of *CXCL10*^−/−^ mice. WT and *CXCL10*^−/−^ mice were fed either chow or FFC diet. (**a**) Absolute body weight and body weight gain in gram (**g**) over the study period, (**b**) weight ratio of lean mass (%) and fat mass (%). (**c**) Energy expenditure (kcal/hour/kg). (**d**) Physical activity (counts). (**e**) Respiratory quotient. (**f**) HOMA-IR calculated from fasting glucose and insulin levels. (**g**) Glucose tolerance test and area under the curve calculated based on the serial measurements of blood glucose concentrations at fasting, and then 15, 30, 60, 90, and 120 minutes after a 1.5 gram/kg glucose bolus administration. Bar columns represent mean ± standard error of the mean. **p <  0.01; *p < 0.05; ns (non-significant).

Total body weights were similar between *CXCL10*^−/−^ and wild type (WT) mice on standard chow and the high saturated fat, fructose, and cholesterol (FFC) diet (Fig. 1a). The FFC diet caused significantly greater gains in body weight than standard chow in both *CXCL10*^−/−^ and WT mice (Fig. 1a), primarily due to increases in fat mass (Fig. 1b). No differences in body composition (percent of lean mass and fat mass) were observed between *CXCL10*^−/−^ and WT mice on standard chow and the FFC diet (Fig. 1b). Moreover, caloric intake (data not shown), energy expenditure (Fig. 1c), physical activity (Fig. 1d) and respiratory quotient (Fig. 1e) were comparable between FFC-fed *CXCL10*^−/−^ and WT mice. Insulin resistance was observed in FFC-fed WT mice and *CXCL10*^−/−^ mice as assessed by the Homeostasis model assessment of insulin resistance (HOMA-IR). Interestingly*, CXCL10*^−/−^ mice had a higher HOMA-IR (Fig. 1f), secondary to higher fasting insulin level. The fasting blood glucose concentrations were similar in WT and *CXCL10*^−/−^ mice. This observation might be attributed to the pathogenic role of CXCL10 in pancreatic islet β cell dysfunction^21^,^22^, and to the significantly increased level of circulating CXCL10 in FFC-fed mice^12^. The HOMA-IR is consistent with the glucose tolerance test (GTT) that demonstrates similar fasting blood glucose, with more significantly increased excursion of glucose concentrations from baseline during the GTT after the glucose bolus, and more significant area under the curve in the FFC-fed WT mice *vs. CXCL10*^−/−^ mice on the same diet (Fig. 1g). These data suggest that the FFC-fed WT mice have started to develop islet β cell dysfunction, while *CXCL10*^−/−^ mice are relatively protected against islet β cell dysfunction and loss, and therefore have higher insulin levels. Thus, overall the FFC diet induces similar metabolic changes in both *CXCL10*^−/−^ and WT mice in our model.

### FFC-fed WT and CXCL10^−/−^ mice develop similar hepatic steatosis

**Figure 2:**
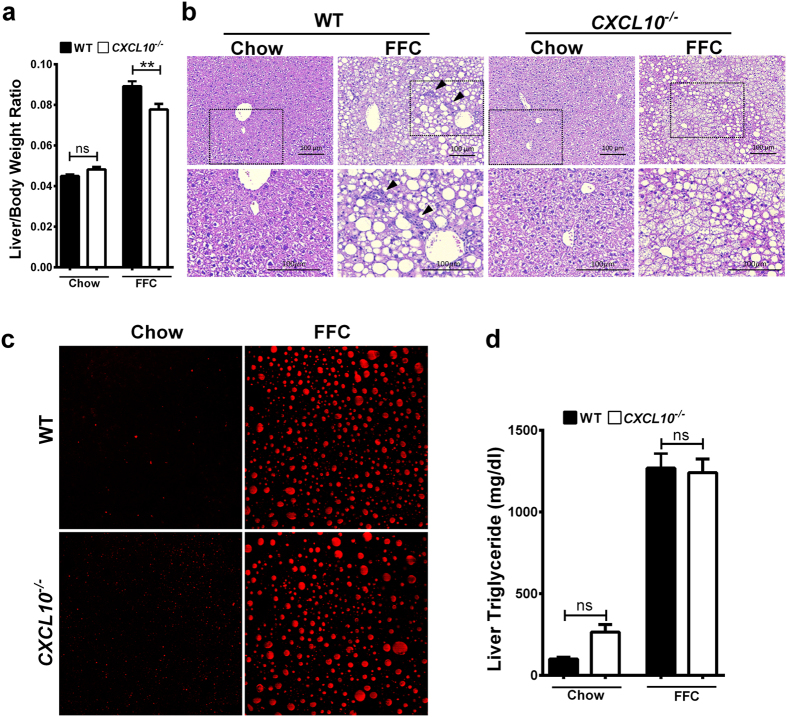
FFC-fed WT and *CXCL10*^−/−^ mice develop similar hepatic steatosis. WT and *CXCL10*^−/−^ mice were fed either chow or FFC diet for 20 weeks. (**a**) Liver/Body weight ratio was calculated. (**b**) Fixed liver tissues from WT and *CXCL10*^−/−^ mice were stained with hematoxylin and eosin (H&E); inflammatory infiltrates were indicated with the black arrowheads. Bottom row contains images enlarged from the boxed area in the corresponding panel in the top row. (**c**) Label-free frozen liver tissue sections were imaged by coherent anti-stokes raman scattering (CARS) microscopy to visualize lipid droplets using a 25× objective. (**d**) Concentration of neutral triglycerides (TG) (mg/dl) was measured by photometric absorbance based technique in the liver tissue of WT and *CXCL10*^−/−^ mice on chow and FFC diet. Bar columns represent mean ± standard error of the mean. **p <  0.01; ns (non-significant).

Although the whole body weight was not different between *CXCL10*^−/−^ and WT mice, FFC-fed *CXCL10*^−/−^ mice have significantly lower liver/body weight ratio compared to WT mice (Fig. 2a). Histological examination of the liver by hematoxylin and eosin (H&E) stain displayed similar extent of steatosis in the FFC-fed WT versus *CXCL10*^−/−^ mice (Fig. 2b). We assessed steatosis by coherent anti-Stokes Raman scattering (CARS) microscopy, which showed similar extent of steatosis in the FFC-fed *CXCL10*^−/−^ and WT mice (Fig. 2c). Likewise, the triglyceride (TG) liver contents were similar in FFC-fed *CXCL10*^−/−^ and WT mice (Fig. 2d). Although, there were prominent inflammatory infiltrates on H&E stain in the WT mice, these infiltrates were quite diminished in the *CXCL10*^−/−^ mice (Fig. 2b). Thus, FFC-fed *CXCL10*^−/−^ mice have similar hepatic steatosis as compared to WT mice on the same obesity-inducing diet, but display reduced hepatic inflammatory infiltrates.

### CXCL10^−/−^ mice are protected against FFC diet-induced macrophage-associated hepatic inflammation

**Figure 3:**
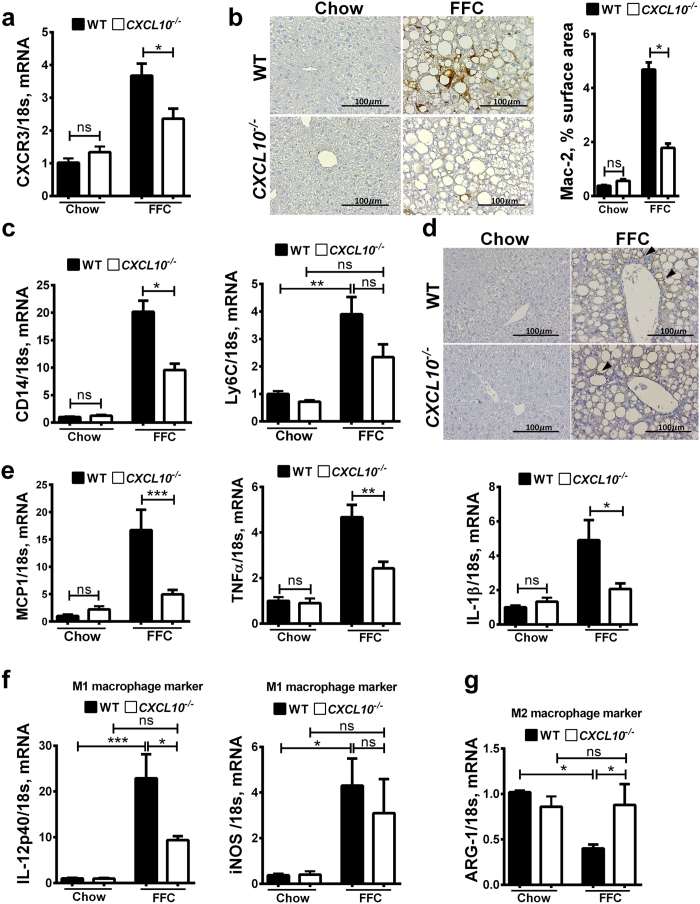
*CXCL10*^−/−^ mice are protected against FFC diet-induced macrophage-related liver inflammation. WT and *CXCL10*^−/−^ mice were fed either chow or FFC diet for 20 weeks. Total RNA was extracted from the liver tissue of wild type (WT) and *CXCL10*^−/−^ mice. The mRNA expression of (**a**) CXCR3, (**b**) Assessment of macrophage infiltration in the WT and *CXCL10*^−/−^ mice on chow and FFC diet was done by immunohistochemistry using macrophage galactose-specific lectin (Mac-2) antibody, Mac-2 immunohistochemical staining was quantified in ten random 20 × microscopic fields per animal by morphometry using ImageJ software, (**c**) the general macrophage surface marker CD14, and the monocyte marker Ly6C were evaluated by real-time PCR. (**d**) Ly6C positive cells were assessed by immunohistochemistry. Ly6C positive cells were stained brown (black arrowheads). (**e**) Cytokines and chemokines related to macrophage activation including MCP-1, TNFα, and IL-1β were evaluated by real-time PCR. (**f**) M1 macrophage surface markers IL-12p40 and iNOS, and (**g**) M2 macrophage surface marker ARG-1 were also evaluated by real-time PCR. Fold induction was determined after normalization to 18s mRNA expression in liver tissue of WT and *CXCL10*^−/−^ mice and expressed relative to that observed in chow-fed WT mice. Bar columns represent mean ± standard error of the mean. ***p < 0.001; **p < 0.01; *p < 0.05; ns (non-significant).

We examined CXCL10-mediated trafficking of various innate immune cells to the liver in our NASH mouse model. We first assessed the expression of CXCR3 (the cognate receptor of CXCL10) in whole liver by real-time PCR. Interestingly, CXCR3 mRNA hepatic expression was significantly reduced in FFC-fed *CXCL10*^−/−^ mice *vs*. WT mice (Fig. 3a), suggesting that loss of the potent chemotactic ligand CXCL10 results in reduced trafficking to the liver of the CXCR3 expressing innate immune cells in general and macrophages in particular. We next, profiled the different innate immune cell populations involved in the sterile inflammatory response observed in our NASH mouse model. We assessed hepatic macrophage infiltration by immunohistochemistry for macrophage galactose specific lectin (Mac-2) (a marker of phagocytically active macrophages)^23^. The FFC diet caused an increase in the Mac-2 positive stained surface area, which was significantly reduced in FFC-fed *CXCL10*^−/−^ vs. WT mice (Fig. 3b). Likewise, FFC-fed mice had a significant increase in the mRNA expression of the general macrophage surface marker cluster of differentiation (CD) 14 24, while the loss of CXCL10 significantly reduced CD14 hepatic expression (Fig. 3b). Similarly, the FFC diet caused an increase in the mRNA expression of lymphocyte antigen 6 complex (Ly6C) (an inflammatory monocyte-derived macrophage marker)^25^ to a more significant extent in the WT vs. *CXCL10*^−/−^ mice (Fig. 3c). Likewise, CXCL10 deletion reduced FFC diet-induced hepatic infiltration with Ly6C positive cells as assessed by immunohistochemistry (Fig. 3d). Furthermore, mRNA expressions of the macrophage activation markers TNFα, IL-1β and MCP-1 were increased by the FFC diet, but significantly reduced in the FFC-fed *CXCL10*^−/−^ vs. WT mice (Fig. 3e). In addition, mRNA expression of IL-12p40 and inducible nitrous oxide synthase (iNOS), known M1 (proinflammatory) markers^26^ were reduced in FFC-fed *CXCL10*^−/−^ mice vs. WT mice (Fig. 3f). Moreover, mRNA expression of arginase (ARG)-1, a known M2 (anti-inflammatory) marker^27^, was reduced with the FFC diet in the WT but not the *CXCL10*^−/−^ mice (Fig. 3g). Taken together, these data demonstrate reduced proinflammatory macrophage hepatic infiltration and activation in the FFC-fed *CXCL10*^−/−^ mice.

**Figure 4:**
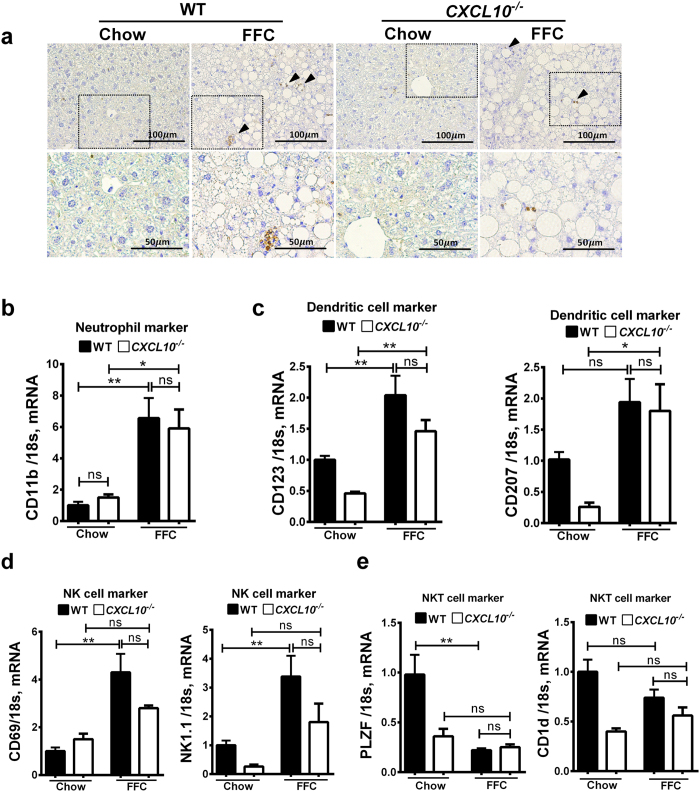
Innate immune cells are overall increased in FFC-fed mice. WT and *CXCL10*^−/−^ mice were fed either chow or FFC diet for 20 weeks. (**a**) Fixed liver tissue sections were stained for myeloperoxidase to detect neutrophil hepatic infiltration (black arrowheads show stain positive cells). Bottom row contains images enlarged from the boxed area in the corresponding panel in the top row. Total RNA was extracted from the liver tissue of wild type (WT) and *CXCL10*^−/−^ mice, and mRNA expression of (**b**) CD11b a neutrophil marker, (**c**) CD123 and CD207 (dendritic cell markers), (**d**) CD69 and NK1.1 (natural killer cell markers), (**e**) PLZF and CD1d (natural killer T-cell markers) were evaluated by real-time PCR. Fold induction was determined after normalization to 18 s mRNA expression in liver tissue of WT and *CXCL10*^−/−^ mice and expressed relative to that observed in chow-fed WT mice. Bar columns represent mean ± standard error of the mean. **p < 0.01; *p < 0.05; ns (non-significant).

We next examined the contribution of other innate immune cells (including neutrophils, dendritic cells, NK cells and NKT cells) to the sterile inflammatory response observed in our NASH mouse model. To assess neutrophil hepatic infiltration, immunohistochemistry for myeloperoxidase (MPO) was performed on hepatic histological sections of mice from the four different experimental groups. The MPO positive stained cells were increased with the FFC diet, and were more prominent in the WT *vs. CXCL10*^−/−^ mice (Fig. 4a). Similarly, the FFC diet caused an increase in the mRNA expression of CD11b (a known neutrophil marker)^25^ to a more significant extent in the WT *vs. CXCL10*^−/−^ mice (Fig. 4b). Dendritic cell hepatic infiltration was examined by assessing mRNA expressions of the known dendritic cell markers CD123 28 and CD207 29, which were increased to a similar extent in the FFC diet in both *CXCL10*^−/−^ and WT mice (Fig. 4c). CD69 and NK1.1 were employed as markers of NK cells^25^, and their mRNA levels were significantly increased in WT FFC-fed mice *vs*. chow-fed mice, although their expression was not statistically different in FFC-fed *CXCL10*^−/−^ mice compared to WT mice (Fig. 4d). Finally, we employed promyelocytic leukemia zinc finger (PLZF) and CD1d as NKT cell markers^25^,^30^. The mRNA expression of these genes displayed a similar trend of reduction in WT and *CXCL10*^−/−^ FFC-fed mice compared to chow-fed mice (Fig. 4e), as previously described in obese mice^19^. Collectively, these data suggest that hepatic infiltrations with neutrophils, dendritic cells, and NK cells are all increased in diet-induced NASH in WT mice, without a significant differential regulation in the *CXCL10*^−/−^ mice; albeit, a trend was observed for CXCL10-dependent accumulation of NK cells in the liver of FFC-fed animals.

### CXCL10^−/−^ mice are protected against FFC diet-induced liver injury and fibrosis

**Figure 5:**
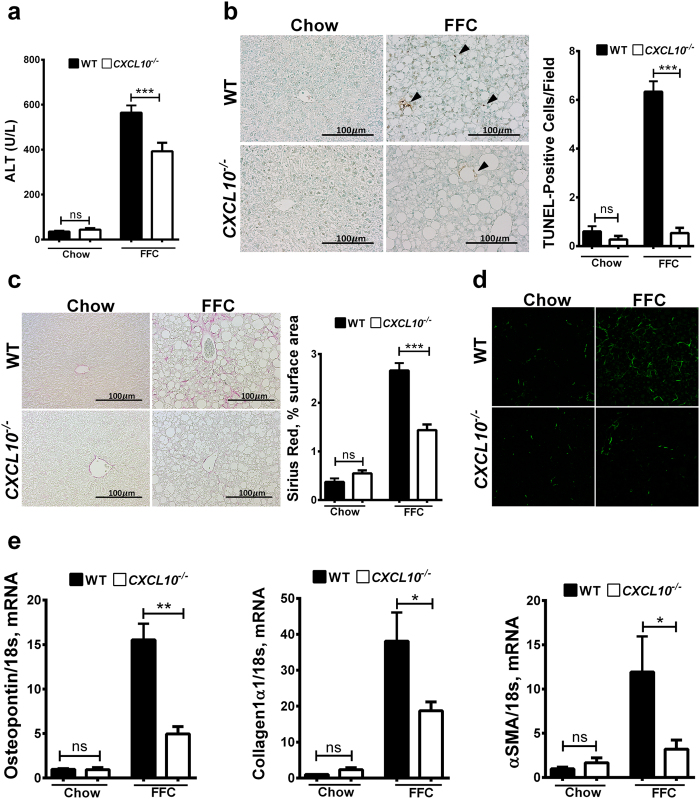
CXCL10^−/−^ mice are protected against FFC diet-induced liver injury and fibrosis. WT and *CXCL10*^−/−^ mice were fed either chow or FFC diet for 20 weeks (**a**) Serum ALT (U/L) values measured by a veterinary chemistry analyzer. (**b**) Hepatocytes apoptosis was quantified in paraffin-embedded hepatic tissue by labeling DNA strand breaks by the terminal deoxynucleotidyl transferase-mediated deoxyuridine triphosphate nick-end labeling (TUNEL) assay. Apoptotic nuclei were stained brown (black arrowheads). TUNNEL-positive nuclei were quantified by counting nuclei in 5 random 20 × microscopic fields per animal. (**c**) Fixed liver tissue sections were stained with Sirius red staining to detect collagen deposition. Quantification of Sirius red chromogen in ten random 20 × microscopic fields per animal was done by morphometry using ImageJ software. (**d**) Label-free frozen liver tissue sections were imaged by second harmonic generation (SHG) microscopy to visualize collagen deposition using a 25× objective. Total RNA was extracted from the liver tissue of wild type (WT) and *CXCL10*^−/−^ mice and mRNA expression of (**e**) the profibrogenic markers osteopontin, collagen 1α1, and αSMA were evaluated by real-time PCR. Fold induction was determined after normalization to 18 s mRNA expression and expressed relative to that observed in chow-fed WT mice. Bar columns represent mean ± standard error of the mean. ***p < 0.001; **p < 0.01; *p < 0.05; ns (non-significant).

*CXCL10*^−/−^ mice on the FFC diet had significantly lower serum alanine aminotransferase (ALT) levels, consistent with reduced liver injury (Fig. 5a). Likewise, *CXCL10*^−/−^ mice were relatively protected against FFC diet-induced lipoapoptosis as demonstrated by a significant decrease in the number of terminal deoxynucleotidyl transferase-mediated deoxyuridine triphosphate nick-end labeling (TUNEL) positive cells in *CXCL10*^−/−^ mice vs. WT mice (Fig. 5b). Given the fact that liver fibrosis is a marker of disease progression and severity and a consequence of active inflammation and liver injury, we examined the role of CXCL10 in FFC diet-induced NASH related fibrosis. We performed Sirius red stain on liver section. Sirius red-positive surface area was significantly reduced in FFC-fed *CXCL10*^−/−^ vs. WT mice (Fig. 5c). These findings were confirmed by second harmonic generation (SHG) microscopy for collagen fibrils (Fig. 5d). Furthermore, we examined the expression of fibrosis-related genes, including osteopontin, collagen 1a1, and α smooth muscle actin (αSMA). The mRNA levels of all these genes were increased by the FFC diet, with a significant reduction in the *CXCL10*^−/−^ mice vs. WT mice (Fig. 5e). Thus *CXCL10*^−/−^ mice appear to be protected against FFC diet-induced hepatic injury and fibrosis.

### CXCR3^−/−^ mice display reduced FFC-induced liver inflammation, injury and fibrosis

**Figure 6:**
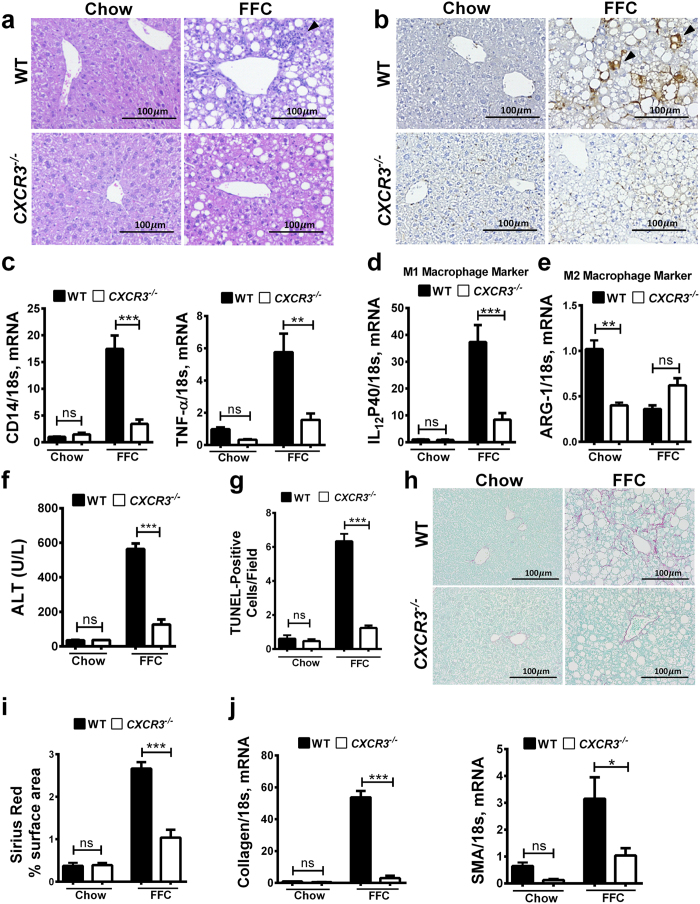
*CXCR3*^−/−^ mice have reduced FFC-induced liver injury, inflammation and fibrosis. WT and *CXCR3*^−/−^ mice were fed either chow or FFC diet for 20 weeks. (**a**) Fixed liver tissue sections were stained with hematoxylin & eosin (H&E); inflammatory infiltrates were indicated with the black arrowheads. (**b**) Macrophages infiltration was assessed by immunohistochemistry using macrophage galactose-specific lectin (Mac-2) antibody; macrophage infiltrates were indicated with the black arrowheads. Total RNA was extracted from the liver tissue of wild type (WT) and *CXCR3*^−/−^ mice, and (**c**) mRNA expression of the general macrophage surface marker CD14, and the macrophage activation markers TNFα, (**d**) the M1 macrophage marker IL-12p40 and (**e**) the M2 macrophage marker arginase were evaluated by real-time PCR. (**f**) Serum ALT (U/L) values were measured by a veterinary chemistry analyzer. (**g**) Hepatocytes apoptosis was quantified in paraffin-embedded hepatic tissue by labeling DNA strand breaks by the terminal deoxynucleotidyl transferase-mediated deoxyuridine triphosphate nick-end labeling (TUNEL) assay. (**h**) Fixed liver tissue sections were stained with Sirius red staining to detect collagen deposition. (**i**) Quantification of Sirius red chromogen in ten random 20 × microscopic fields per animal was done by morphometry using ImageJ software. (j) mRNA expression of profibrogenic markers collagen 1α1, and αSMA were evaluated by real-time PCR. Fold induction was determined after normalization to 18 s mRNA expression in liver tissue of WT and *CXCR3*^−/−^ mice and expressed relative to that observed in chow-fed WT mice. Bar columns represent mean ± standard error of the mean. ***p < 0.001; **p < 0.01; ns (non-significant).

To further demonstrate the role of CXCL10 in hepatic inflammation in this model, mice genetically deficient in the CXCL10 cognate receptor, CXCR3, were placed on the FFC diet. FFC-fed *CXCR3*^−/−^ mice displayed reduced liver injury when compared to WT mice on the same diet as demonstrated by a more favorable histology (Fig. 6a) with less inflammatory infiltrates. These mice also had reduced hepatic macrophage infiltration, as defined by Mac-2 immunohistochemistry (Fig. 6b), and hepatic mRNA expression of the general macrophage surface marker CD14 (Fig. 6c). In addition, these mice had reduced macrophage activation marker TNFα (Fig. 6c) and M1 macrophage infiltration, as assessed by the mRNA expression of IL-12p40 (Fig. 6d), while the mRNA expression of the M2 macrophage marker arginase was not reduced in the FFC-fed *CXCR3*^−/−^ mice vs. WT mice (Fig. 6e). Furthermore, FFC-fed *CXCR3*^−/−^ mice have reduced liver injury, as assessed by serum ALT values (Fig. 6f), and the number of apoptotic hepatocytes when compared to WT mice on the same diet (Fig. 6g). Likewise, when compared to WT mice FFC-fed *CXCR3*^−/−^ mice have reduced liver fibrosis, as assessed by Sirius red stain (Fig. 6h) and mRNA expression of αSMA and collagen 1a1 (Fig. 6j). Taken together, these data demonstrate that genetic deletion of CXCR3 is also protective against a nutrient excess diet-induced NASH in mice.

## Discussion

The principal findings of the present study provide mechanistic insights regarding the protective effect of CXCL10 genetic deletion in a dietary model of NASH. Our data indicate that CXCL10 genetic deletion conveys several salutary effects that are principally mediated by diminished hepatic macrophage trafficking to the liver, infiltration and activation with subsequent decrease in hepatic injury and fibrosis; furthermore, we substantiate our findings and demonstrate that genetic deletion of the CXCL10 cognate receptor CXCR3 reduces diet-induced murine NASH. These observations are independent of the obesity and the metabolic changes generated by the FFC diet and are more thoroughly discussed below.

In this study, we used a well-established dietary model of NASH in the mouse^29^. The model includes a diet high in saturated fats, cholesterol and the addition of fructose in the drinking water, and hence is termed the FFC diet for Fat, Fructose, and Cholesterol. The FFC diet was developed to replicate the western fast food diet and phenocopies the histological features of human NASH including neutral lipid accumulation by hepatocytes, the presence of ballooned hepatocytes, hepatic inflammatory cells infiltration, and liver fibrosis. The model has a high fidelity to the metabolic profile observed in humans, including obesity, hyperlipidemia and insulin resistance^12^,^31^. Indeed, FFC-fed *CXCL10*^−/−^ and WT mice had insulin resistance, similar weight gain and metabolic profiles. Insulin resistance is both peripheral and hepatic in our current model. WT and *CXCL10*^−/−^ mice on the FFC diet had similar increase in hepatic steatosis and triglyceride content. A growing body of evidence suggests that hepatic triglyceride accumulation contributes to impaired glucose metabolism and insulin sensitivity in muscle and in the liver^32^,^33^,^34^.

Zhang *et al*. have recently reported that methionine and choline deficient (MCD) diet-fed *CXCL10*^−/−^ mice have reduced liver steatosis, injury, inflammation, and fibrosis^35^. We made similar observations in our study regarding the protective effect of *CXCL10*^−/−^ against liver injury and fibrosis; although, in our current study, we did not observe a difference in hepatic steatosis and TG content between the FFC-fed *CXCL10*^−/−^ and WT mice. Although the MCD diet replicates the histological features of steatohepatitis and fibrosis observed in human NASH, its metabolic context is distinct from human NASH, since animal fed the MCD diet lose up to 40% of their weight in 10 weeks, have low fasting blood sugar, peripheral insulin sensitivity, low serum insulin, decreased blood triglyceride and cholesterol^36^,^37^. This metabolic profile is quite opposite to human obesity-associated NASH, which may explain the discrepancy observed in hepatic steatosis between the MCD-fed *CXCL10*^−/−^ mice in the study by Zhang *et al*. vs. FFC-fed *CXCL10*^−/−^ mice in the current study. Furthermore, in the current study, we implicate macrophage induced hepatic inflammation as a key player in CXCL10-mediated murine NASH. Chronic activation of the innate immune cells is an essential feature of NASH. A growing body of evidence suggests that Kupffer cells and recruited macrophages/monocytes play a major role in NASH pathogenesis^10^,^11^. We have previously confirmed that macrophages express CXCR3, the CXCL10 cognate receptor. Furthermore, using a trans-well migration assay, we demonstrated that CXCL10 is a potent chemotactic ligand for macrophages, which chemotaxis was abolished with the addition of the CXCL10 blocking antibody^12^. To define the major players in the inflammatory response observed in our *in vivo* model, we profiled the hepatic infiltration of the different subpopulations of the innate immune system. Interestingly, FFC-fed *CXCL10*^−/−^ mice have reduced monocyte derived macrophage infiltration in general and macrophage with the M1 phenotype in particular, likely secondary to reduced total number of macrophages. These observations substantiate the role of CXCL10 in mediating macrophage trafficking to the liver in murine NASH. Furthermore, we observed a reduction in the FFC diet-induced macrophage activation in the *CXCL10*^−/−^ mice, as assessed by decreased hepatic expression of macrophage-derived proinflammatory cytokines and chemokines.

Although, neutrophil, DC, NK cells were all significantly increased with the FFC diet, in contrast to macrophages, significant differences were not observed for their corresponding markers between FFC-fed WT and *CXCL10*^−/−^ mice. Similar to a previous report, NKT cells displayed an opposite behavior, when compared to other immune cells, in response to the FFC diet in that their hepatic number are reduced in obesity^19^.

Different innate immune cells involved in NASH pathogenesis express CXCR3, the cognate receptor of CXCL10. Interestingly, CXCR3 expression was significantly reduced in *CXCL10*^−/−^ mice, supporting our hypothesis that loss of CXCL10 results in reduced trafficking of the CXCR3 expressing innate immune cells in general and macrophages in particular to the liver, resulting in significant attenuation of the hepatic inflammation observed in FFC-fed *CXCL10*^−/−^ mice. We further confirmed this observation by demonstrating a protective effect of CXCR3 genetic deletion against FFC diet-induced liver inflammation, injury and fibrosis. It is to be noted that CXCR3 is the cognate receptor of the chemotactic ligands CXCL9 and CXCL11 in addition to CXCL10; these chemokines may have some redundant effect with CXCL10^38^, and explain the more significant attenuation of the FFC diet-induced NASH in the *CXCR3*^−/−^ mice when compared to the *CXCL10*^−/−^ mice.

In summary, we demonstrate a salutary effect of CXCL10 genetic deletion in a preclinical model of NASH pathogenesis independent of body weight and the metabolic syndrome. Although CXCR3 is expressed in all the innate immune cells, macrophage-associated inflammation appears to be the lynch pin for the CXCL10-mediated sterile inflammatory response observed in murine NASH. We speculate that the other innate immune cells have an alternative mechanism of chemotaxis independent of CXCL10. Given the protective effect of the CXCL10 and CXCR3 genetic deletion in our murine dietary model of NASH, pharmacological inhibitions of CXCL10 or CXCR3 are potential therapeutic strategies to attenuate the sterile inflammatory response in human NASH.

## Methods

### Animals

Study protocols were conducted as approved by the Institutional Animal Care and Use Committee of Mayo Clinic. The methods employed in the current study were carried out in accordance with IACUC guidelines for the use of anesthetics in experimental mice. We bred homozygous WT, *CXCL10*^−/−^ and *CXCR3*^−/−^ mice. Mice were acquired from Jackson Laboratory, (ME, USA). *CXCL10*^−/−^ and *CXCR3*^−/−^ mice shared the same genetic background C57BL/6J as WT mice, and were weight and age matched at the initiation of the study, housed and fed under the same environmental conditions. The animals were randomized to standard chow (Teklad-Harlan, WI, USA), or FFC diet plus fructose added to the drinking water (42 g/l final concentration) as previously described in detail^31^. This model of diet-induced obesity displays high fidelity to human NASH, and results in the development of murine NASH with liver inflammation, ballooned hepatocytes, and bridging fibrosis^23^,^31^. Seven mice were used in each of the experimental groups (chow-WT, FFC-WT, chow-knock out, FFC-knock out). Animals were provided free access to diet for 20 weeks. At 14 weeks, metabolic parameter including food intake, oxygen consumption, carbon dioxide production, and locomotor activity were measured using a Comprehensive Laboratory Animal Monitoring System (Columbus Instruments, OH, USA) as previously described^39^. Blood glucose was measured using a blood glucose monitor (Assure 4, Arkray, MN, USA) and plasma insulin was measured by Ultra-Sensitive Mouse Insulin Enzyme-Linked Immuno Sorbent Assay (ELISA) kit (Crystal Chem. Inc., IL, USA) as per the manufacturer’s instructions. HOMA-IR was calculated by using the following formula: HOMA-IR = 26× fasting insulin level (ng/ml) × fasting glucose level (mg/dl)/405 40. Glucose tolerance test (GGT) was performed by serial measurement of mice blood glucose at fasting status, and then at 15, 30, 60, 90 and 120 minutes after intraperitoneal administration of a glucose bolus of 1.5 gram/kg. Mice were sacrificed under general anesthesia induced by ketamine/xylazine cocktail (83 mg/kg ketamine/16 mg/kg xylazine, intraperitoneal). Blood and liver samples were collected for further analysis.

### Liver triglyceride and serum alanine aminotransferase quantification

Liver TG levels were measured from mouse liver homogenates. Briefly, 50 milligrams of wet liver tissue were homogenized in a 5% NP-40 solution. EnzyChrom Triglyceride Kit (BioAssay System, CA, USA) was used for the assay according to manufacturer’s instructions. Photometric absorbance was read at 570 nm using Synergy H1 microplate reader (BioTek, VT, USA). ALT levels were measured using a veterinary chemistry analyzer (VetScan VS2, CA, USA).

### Histology, immunohistochemistry, and digital image analysis

Liver histology was performed using tissue fixed in 10% formalin, dehydrated and embedded in paraffin. Sections were stained with H&E, and Sirius red stain. TUNEL-positive cells were quantified using the Apop Tag Peroxidase *in Situ* Apoptosis Detection Kit (Millipore, MA, USA), diaminobenzidine (DAB) was used as a peroxidase substrate (Vector Laboratories, CA, USA); 0.5% methyl green was used for the counterstain. For immunohistochemistry, paraformaldehyde-fixed paraffin-embedded liver tissue sections were deparaffinized, hydrated and stained with antibody against Mac-2 (1:250, eBioscience, CA, USA), Ly6C (1:1000, abcam, Cambridge, MA, USA) and MPO (3 μg/mL, R&D systems, MN, USA). Bound antibodies were detected using Vectastain ABC kit (Vector Laboratories, CA, USA) and DAB stain; the tissue sections were counterstained with hematoxylin. Sirius red-stained area and Mac-2 positive area were quantified by digital image analysis of five random fields per slide using the ImageJ software (NIH, MD, USA). Frozen liver sections were imaged on a two photon confocal microscope FluoView FV1000 MPE (Olympus America, Center Valley, PA) using the CARS microscopy application to identify lipid droplet within hepatocytes, and SHG application to identify collagen deposition. Mai Tai Deep Sea laser (Spectra-Physics, CA, USA) was tuned to 800 nm and an XLPlanN 256/1.05w MP objective lens was used, as described by us^41^.

### Quantitative real-time polymerase chain reaction (PCR)

Total RNA was isolated with RNeasy Mini Kit (Qiagen, CA, USA) and was reverse transcribed with moloney murine leukemia virus reverse transcriptase and oligo-dT random primers (both from Invitrogen, CA, USA). Quantification of gene expression was performed by real-time PCR using SYBR green fluorescence on a Light Cycler 480 instrument (Roche Applied, IN, USA). Primers are listed in Table 1. Target gene expression was calculated using the ΔΔCt method and expression was normalized to 18 s rRNA expression levels.

### Statistical analysis

Data are expressed as the means ± SEM. Differences between multiple groups were compared using one-way analysis of variance followed by Bonferroni’s multiple comparisons test. *, **, ***, indicate statistical significance with p < 0.05, p < 0.01 and p < 0.001 respectively. Statistically non-significant results were labeled as ns where appropriate. All analyses were performed using GraphPad Prism 6 software (CA, USA).

## Additional Information

**How to cite this article**: Tomita, K. *et al*. CXCL10-Mediates Macrophage, but not Other Innate Immune Cells-Associated Inflammation in Murine Nonalcoholic Steatohepatitis. *Sci. Rep*. **6**, 28786; doi: 10.1038/srep28786 (2016).

## Figures and Tables

**Table 1:** Mouse primers used for qPCR.

Gene	Forward primer sequence (5′-3′)	Reverse primer sequence (5′-3′)
CXCR3	TACCTTGAGGTTAGTGAACGTCA	CGCTCTCGTTTTCCCCATAATC
CD14	CTCTGTCCTTAAAGCGGCTTAC	GTTGCGGAGGTTCAAGATGTT
MCP-1	GCATTAGCTTCAGATTTACGGG	GCATTAGCTTCAGATTTACGGG
TNFα	CCCTCACACTCAGATCATCTTCT	GCTACGACGTGGGCTACAG
IL-1β	GCAACTGTTCCTGAACTCAACT	ATCTTTTGGGGTCCGTCAACT
iNOS	TTCACCCAGTTGTGCATCGACCTA	TCCATGGTCACCTCCAACACAAGA
IL-12p40	TGGTTTGCCATCGTTTTGCTG	ACAGGTGAGGTTCACTGTTTCT
ARG-1	CTCCAAGCCAAAGTCCTTAGAG	AGGAGCTGTCATTAGGGACATC
CD11b	ATGGACGCTGATGGCAATACC	TCCCCATTCACGTCTCCCA
Ly6C	GCAGTGCTACGAGTGCTATGG	ACTGACGGGTCTTTAGTTTCCTT
CD123	CTGGCATCCCACTCTTCAGAT	GGTCCCAGCTCAGTGTGTA
CD207	CCGAAGCGCACTTCACAGT	GCAGATACAGAGAGGTTTCCTCA
CD69	TGGTCCTCATCACGTCCTTAATAA	TCCAACTTCTCGTACAAGCCTG
NK1.1	TCATCCTCCTTGTCCTGA CC	TTGAATGAGCAGCAAAGTGG
PLZF	CTGCGGAAAACGGTTCCTG	GTGCCAGTATGGGTCTGTCT
CD1d	AATCTGAAGCCCAGCAAAAGAA	TTACTCCAACGGTGAGTCTGC
Osteopontin	CTCCATCGTCATCATCATCG	TGCACCCAGATCCTATAGCC
Collagen1a1	GCTCCTCTTAGGGGCCACT	CCACGTCTCACCATTGGGG
αSMA	GTCCCAGACATCAGGGAGTAA	TCGGATACTTCAGCGTCAGGA
18 s	CGCTTCCTTACCTGGTTGAT	GAGCGACCAAAGGAACCATA
